# Disentangling the Environmental Factors That Shape Genetic and Phenotypic Leaf Trait Variation in the Tree *Qualea grandiflora* Across the Brazilian Savanna

**DOI:** 10.3389/fpls.2019.01580

**Published:** 2019-12-02

**Authors:** Renata Santiago de Oliveira Buzatti, Thais Ribeiro Pfeilsticker, André Carneiro Muniz, Vincenzo A. Ellis, Renan Pedra de Souza, José Pires Lemos-Filho, Maria Bernadete Lovato

**Affiliations:** ^1^Laboratório de Genética de Populações, Departamento de Genética, Ecologia e Evolução, Instituto de Ciências Biológicas, Universidade Federal de Minas Gerais, Belo Horizonte, Brazil; ^2^Molecular Ecology and Evolution Lab, Department of Biology, Lund University, Lund, Sweden; ^3^Department of Entomology and Wildlife Ecology, University of Delaware, Newark, DE, United States; ^4^Grupo de Pesquisa em Bioestatística e Epidemiologia Molecular, Departamento de Genética, Ecologia e Evolução, Universidade Federal de Minas Gerais, Belo Horizonte, Brazil; ^5^Laboratório de Fisiologia Vegetal, Departamento de Botânica, Instituto de Ciências Biológicas, Universidade Federal de Minas Gerais, Belo Horizonte, Brazil

**Keywords:** cerrado, climate, genetic divergence, isolation by distance, leaf traits diversity, *Qualea grandiflora*

## Abstract

Identifying the environmental factors that shape intraspecific genetic and phenotypic diversity of species can provide insights into the processes that generate and maintain divergence in highly diverse biomes such as the savannas of the Neotropics. Here, we sampled *Qualea grandiflora*, the most widely distributed tree species in the Cerrado, a large Neotropical savanna. We analyzed genetic variation with microsatellite markers in 23 populations (418 individuals) and phenotypic variation of 10 metamer traits (internode, petiole and corresponding leaf lamina) in 36 populations (744 individuals). To evaluate the role of geography, soil, climate, and wind speed in shaping the divergence of genetic and phenotypic traits among populations, we used Generalized Dissimilarity Modelling. We also used multiple regressions to further investigate the contributions of those environmental factors on leaf trait diversity. We found high genetic diversity, which was geographically structured. Geographic distance was the main factor shaping genetic divergence in *Qualea grandiflora*, reflecting isolation by distance. Genetic structure was more related to past climatic changes than to the current climate. We also found high metamer trait variation, which seemed largely influenced by precipitation, soil bulk density and wind speed during the period of metamer development. The high degree of metamer trait variation seems to be due to both, phenotypic plasticity and local adaptation to different environmental conditions, and may explain the success of the species in occupying all the Cerrado biome.

## Introduction

Understanding the role of environmental factors in shaping genetic and phenotypic intraspecific diversity can provide important insights into the processes that are responsible for the generation and maintenance of biological diversity (Ribeiro et al., 2016b; Ji et al., 2017; Oliveira et al., 2018; Souza et al., 2018). In response to environmental factors such as climate and soil, populations can develop local adaptation, a process that results in changes in the genetic composition of populations over multiple generations (Lajoie and Vellend, 2018). Local adaptation can lead to a phenomenon known as isolation by environment – IBE, the association between genetic divergence among populations and environmental differentiation, independent of geographic distance (Sexton et al., 2013; Wang and Bradburd, 2014). However, genetic divergence can also emerge from a restriction of gene flow between populations promoted by geographic distance (isolation by distance – IBD – Wright, 1943; Slatkin, 1993). Phenotypic divergence in traits can be a result of a genetic divergence due to local adaptation combining with genetic drift and restriction to gene flow. Variation in functional traits have been associated with climate in several biomes around the world (Wright et al., 2004; Ribeiro et al., 2016b; Wright et al., 2017). A well-known trait associated with different climates is the specific leaf mass (SLM), defined as the ratio of dry leaf mass too leaf area. Compared to plants from wetter climates, plants from drier environments exhibit thicker leaves with higher leaf and cell wall thickness, resulting in higher SLM (Niinemets, 2001; Wright et al., 2004; Ribeiro et al., 2016b). High SLM has been associated with longer leaf life under dry conditions in evergreen species (Wright et al., 2004). Leaves with smaller SLM or higher specific leaf area (SLA – area per mass ratio) show less investment in producing non-photosynthesizing structures and shorter leaf lifespans. SLA has been associated with a tradeoff between carbon uptake by photosynthesis and water loss by transpiration, which determine resource-use efficiency and tolerance to environmental stresses, mainly to water stress (Ackerly et al., 2000; Poorter 2009). Besides climate, SLA has been associated with soil conditions, decreasing with soil pH and bulk density (Maire et al., 2015). The leaf length/width ratio (LLW) has also been reported to vary along climatic gradients, increasing with rainfall (Jacobs, 1999). Besides variation in the leaf blade, variation in metamer traits (internode, petiole and corresponding leaf lamina) has been analyzed due to its relevance to function. For example, internode and petiole length and mass are important for the support of leaf lamina with regard to spatial positioning, biomechanics and the hydraulic pathway (Santiago et al., 2004). Lower investment in leaf blade per total mass of metamer and lower specific petiole length have been found to be associated with more arid climate in Cerrado tree species (Ribeiro et al., 2016b; Souza et al., 2018).

Species with broad geographic distributions in heterogeneous environments constitute good models to investigate the role of environmental factors in shaping genetic and phenotypic diversity. In this context, the largest Neotropical savanna, the Cerrado, provides an ideal opportunity to conduct such research since it comprises one of the most biodiverse and heterogeneous tropical biomes on the world. The Cerrado harbors almost 10,000 plant species, and comprises a landscape marked by phytophysiognomies ranging from grassy fields to dense gallery forests (Oliveira and Marquis, 2002; Ratter et al., 2003). The biome extends across a range of 20° latitude in Brazil, encompassing geomorphic surfaces of vast plateaus and wide altitudinal variation (sea level to 1800 m) (Ratter et al., 2003). The Cerrado climate is characterized by two well-marked seasons: a dry season during the southern winter (April to September), and a rainy season (October to March) when most annual precipitation and the highest temperatures occur (Pennington et al., 2006). The soils are mostly of low fertility, acidic, and have high levels of aluminum (Furley and Ratter, 1988). Soil characteristics such as fertility gradients, aluminum content, and water availability have been suggested to determine the diversity and distribution of Cerrado species (Oliveira-Filho and Ratter, 2002; Ratter et al., 2003; Assis et al., 2011; Bueno et al., 2018). Due to the broad variation in climatic and soil conditions and to the large area of the biome, the study of widely distributed species in this biome can significantly contribute towards disentangling the climatic, edaphic and geographic drivers of genetic and phenotypic intraspecific diversity.

The Quaternary climatic oscillations have been recognized as playing a role in shaping the levels and spatial distribution of genetic diversity in Cerrado plant species. Phylogeographical and ecological niche modelling studies of Cerrado species have suggested a common pattern of reduction in species ranges during glacial periods, with retractions mainly in their southern extents. During interglacial periods, with wetter climate and higher temperatures, species expanded their distributions (Collevatti et al., 2012; Novaes et al., 2013; Ribeiro et al., 2016a; Buzatti et al., 2017; Buzatti et al., 2018). A few recent studies have suggested that current climatic conditions also influence the intraspecific genetic diversity and structure and metamer traits diversity of Cerrado species (Ribeiro et al., 2016b; Souza et al., 2018). Temperature seasonality and precipitation of the warmest quarter were the main climatic variables associated with genetic divergence among populations (Ribeiro et al., 2016b). Populations that experienced summers with lower precipitation and hotter winters had heavier leaves and lower SLA (Ribeiro et al., 2016b; Souza et al., 2018). Although studies have pointed to the influence of climatic conditions on intraspecific genetic and functional diversity, the topic is still poorly understood in the Cerrado biome. No study has analyzed species that occur across the entire Cerrado, including in disjunct savanna enclaves located in the Amazonian Forest and the Caatinga, where plants face climatic conditions quite different from those found in the central portion of the Cerrado biome. Furthermore, the influence of edaphic conditions on both genetic and phenotypic intraspecific diversity have never been investigated in Cerrado species.

Here, we assessed genetic diversity using microsatellite markers and variation of leaf metamer traits of *Qualea grandiflora* (Vochysiaceae), the most common and widely distributed tree species of the Cerrado (Ratter et al., 2003). We sampled populations occurring across a broad climatic gradient and with different soil conditions, including savannas patches present in the Amazonian Forest and the Caatinga, a xeric vegetation of northeastern Brazil. Our main goal was to determine the relative contribution of geography and environment (climate, soil and wind) in shaping *Q. grandiflora* genetic and leaf metamer trait variation, using Generalized Dissimilarity Modelling. We also assessed the role of specific climate and soil variables in explaining variation of each leaf trait using multiple regressions. Based on the proposed adaptiveness of the leaf traits that we tested here (Wright et al., 2004; Wright et al., 2017), we hypothesized that the environmental variation along the species’ distribution would explain the metamer trait variation better than geographic distance. However, given that microsatellites are assumed to represent neutral genetic variation, we hypothesized that geographic distance would best explain genetic divergence in the species. In addition, we tested the hypothesis that spatial genetic structure would have been influenced by past climatic changes by comparing genetic structure based on microsatellites with that revealed by cpDNA and nDNA sequence data from a previous study (Buzatti et al., 2018). Our assumption is that similar spatial structure would be evidence of the maintenance of gene flow barriers across time mediated by past climatic changes. The understanding of the factors shaping genetic and functional diversity of Cerrado species can contribute towards our ability to predict scenarios about the persistence of species in the face of anthropogenic pressure in the biome and ongoing human caused climate change.

## Materials and Methods

### Study Species

*Qualea grandiflora* is a widespread tree in the Cerrado and occurs exclusively in this biome, leading it to be considered a marker of Brazilian savanna species. The species is 7-12 m tall (Lorenzi, 1992), with simple opposite, coriaceous and petiolate leaves, with a pair of glands at their base (Silva-Junior, 2005). It has yellow flowers with single petals pollinated mainly by hawkmoths and has wind-dispersed seeds (Oliveira et al., 2004). *Q. grandiflora* is adapted to well-drained, mineral-poor and low pH soils and is an aluminium-accumulating species (Haridasan and Araújo, 1988).

### Study Area

To sample most of the natural distribution of *Q. grandiflora*, we conducted an extensive search of its occurrence in phytosociological studies and herbaria databases. Then, we performed large scale sampling, collecting samples between 2°32′S and 24°10′S and between 41°43′W and 63°03′W, from the Cerrado core, peripheral areas and patches of Cerrado within the Amazon forest biome (Amazonian savannas) (**Table 1** and **Supplementary Figure 1**). The samples were collected with at least 5 meters between each other in order to avoid sampling clonal individuals. The average annual precipitation ranged from 880 to 1,828 mm for the populations sampled in Cerrado core and was 1,963 and 2,279 mm for Amazonian enclave populations [Santarém (SAN) and Humaitá (HTA), respectively]. Considering all populations, annual temperature varies from 18.3 to 27.1°C (Fick and Hijmans, 2017).

**Table 1:** Genetic diversity and fixation indexes of *Qualea grandiflora* populations based on nine microsatellites markers.

	Latitude (S) – Longitude (W)	Sample Size	*N*_A_	*A*_R_	*H*_O_	*H*_E_	*F (nfb)*
*Populations – Northern Group (Abbreviation)*							
Araguaína (AGN)	7° 19’ 40" – 48° 14’ 14"	15	7.9	6.2	0.683	0.758	0.000
Alto do Paraíso de Goiás (APG)	14° 06’ 51" – 47° 31’ 27"	20	9.4	6.6	0.717	0.787	0.020
Novo Jardim (NJA)	11° 48’ 28" – 46° 34’ 05"	20	7.7	5.7	0.661	0.744	0.024
Piripiri (PRI)	4° 08’ 24" – 41° 43’ 07"	18	7.1	5.3	0.473	0.696	0.054
Santarém (SAN)	2° 32’ 12" – 54° 54’ 13"	19	6.6	5.1	0.421	0.704	0.058
		*Total*	14.6	12.0	0.623	0.799	
*Populations – Southern Group (Abbreviation)*							
Analândia (ANA)	22° 07’ 41" – 47° 39’ 03"	15	6.3	5.5	0.598	0.719	0.06
Caldas Novas (CAL)	17° 40’ 32" – 48° 45’ 12"	20	9.4	6.9	0.771	0.823	0.018
Campo Grande (CQG)	20° 26’ 34" – 54° 38’ 47"	13	8.3	6.9	0.625	0.823	0.171*
Furnas (FUR)	20° 41’ 00" – 46° 19’ 37"	19	9.7	6.8	0.655	0.803	0.189*
Jaguariaíva (JAG)	24° 10’ 41" – 49° 40’ 08"	12	7.7	6.6	0.694	0.805	0.027
Martinópolis (MTP)	22° 12’ 27" – 51° 05’ 53"	17	9.0	6.8	0.728	0.785	0.017
Selvíria (SEL)	20° 29’ 52" – 51° 32’ 41"	20	8.6	5.9	0.662	0.735	0.021
Serranópolis (SER)	18° 28’ 32" – 52° 05’ 42"	18	9.6	6.6	0.642	0.77	0.088*
		*Total*	18.0	13.7	0.676	0.812	
*Populations – Westhern Group (Abbreviation)*							
Chapada dos Guimarães (CHG)	15° 21’ 51" – 55° 50’ 16"	20	10.9	7.4	0.767	0.818	0.025
Humaitá (HTA)	7° 34’ 01" – 63° 06’ 11"	16	6.7	5.2	0.538	0.692	0.070*
Vilhena (VHA)	12° 17’ 54" – 60° 24’ 31"	20	8.9	6.4	0.625	0.767	0.028
		Total	14.7	13.3	0.651	0.812	
*Populations* – Eastern Group (Abbreviation)							
Cocos (COC)	14° 05’ 01" – 44° 31’ 04"	20	9.8	6.7	0.735	0.804	0.019
Corinto (COR)	18° 22’ 39" – 44° 30’ 09"	20	9.7	6.9	0.738	0.823	0.025
Grão Mogol (GMG)	16° 32’ 31" – 43° 03’ 05"	20	8.2	6.0	0.731	0.788	0.045
João Pinheiro (JPO)	17° 46′ 04" – 46° 10′ 05"	18	9.0	6.6	0.689	0.797	0.052*
Rio das Contas (RCO)	13° 32’ 31" – 41° 51’ 23"	20	6.7	5.1	0.685	0.732	0.022
		*Total*	15.3	12.2	0.717	0.811	
*Populations – Central-western Group (Abbreviation)*							
Nova Xavantina (NXA)	14° 42’ 53" – 52° 21’ 14"	20	10.6	7.2	0.664	0.783	0.097*
Pirinópolis (PIR)	15° 50’ 21" – 48° 54’ 46"	20	10.8	7.7	0.721	0.854	0.029*
		*Total*	14.2	14.0	0.693	0.839	
*Total Mean*			15.4	15.5	0.672	0.815	

^a^N_A_,number of alleles; A_R_, allelic richness; H_O_, observed heterozygosity; H_E_, expected heterozygosity; F(nfb), inbreeding coefficient estimated using the model that include nfb parameters (where n, null alleles, f, inbreeding coefficient and b, genotyping failures); Numbers in bold with an asterisk represent F(nfb) significant values (P < 0.05).

### Population Sampling and DNA Extraction

For the genetic study, we collected young leaves of *ca*. 20 adult individuals per population, from 23 populations, totaling 418 individuals. The distance between sampled populations ranged from 128.9 to 2,397.2 km (**Supplementary Table 1**). The leaves were dried over silica gel and stored at −20°C to prior to DNA extraction. Genomic DNA was isolated using the cetyltrimethylammonium bromide (CTAB) protocol (Doyle and Doyle, 1987). Quantity and quality of DNA was verified using 1% agarose gels and a NanoDrop^®^ spectrophotometer (Thermo Fisher Scientific, Waltham, MA, USA).

### DNA Amplification and Genotyping

Nine microsatellite markers previously developed for the species by Ritter et al. (2012; Qgr01, Qgr03, Qgr04 and Qgr07) and Buzatti et al. (2014; Qgr13, Qgr21, Qgr22, Qgr23, Qgr24) were amplified using different reactions, depending on the primer used. As suggested by the authors, we added fluoresce in each primer described by Ritter et al. (2012) and M13 tails (sequence: 5′-TTTTCCAGTCACGAC-3′) to each forward primer described by Buzatti et al. (2014). We used a total of 13 µl of mix containing 10–20 ng of DNA, 2.6 µl of special IVB buffer [50 mM KCl, 10 mM Tris-HCl (pH 8.4), 0.1% Triton X-100 – Phoneutria], which releases ∼2 mM of Mg^2+^ during the reaction, 0.25 mM of each dNTP, 1 U Taq polymerase (Phoneutria) and 0.05–0.10 µM of each primer, depending on the primer set used [0.10 µM reverse primer and 0.10 µM forward primer to primers described by Ritter et al. (2012) and 0.10 µM reverse primer, 0.05 µM forward primer and 0.10 µM of M13 fluorescently labelled primers described by Buzatti et al. (2014)]. MgCl_2_ was added to reactions in concentrations ranging from 0.25 to 1 mM. The polymerase chain reactions (PCR) were carried out under two distinct sets of conditions, depending on the primer set: (1) for primers described by Ritter et al. (2012), initial denaturation at 94°C for 1 min, followed by 35 cycles of amplification at 94°C for 1 min, specific annealing temperature of each primer for 1 min, extension at 72°C for 1 min and a final extension at 72°C for 30 min; and (2) for primers described by Buzatti et al. (2014), initial denaturation at 94°C for 5 min, 15 cycles of denaturation at 94°C for 30 sec, annealing temperature of each primer for 1 min, extension at 72°C for 1 min, followed by 25 cycles at 89°C for 30 sec, 53°C for 1 min, 72°C for 1 min and a final extension at 72°C for 30 min. Genotyping was performed on an ABI3130XL automated sequencer (Applied Biosystems, Foster City, CA, USA) using HiDi and ROX-500 size standard (GE Healthcare, Diegem, Belgium). The allele screening was performed using Geneious software version 7.1.3 (Biomatters, New Zealand).

### Genetic Data Analyses

Firstly, we verified the presence of null alleles using the Brookfield 1 method with Microchecker version 2.2.3 (van Oosterhout and Hutchinson, 2004). To evaluate the genetic diversity of each population we estimated the number of alleles (*A*), allelic richness (*A*_R_) using a rarefaction method to correct for disparities among sample sizes, the observed (*H*_O_) and expected (*H*_E_) heterozygosities and departure from Hardy–Weinberg equilibrium with ARLEQUIN version 3.5.1.2 (Excoffier and Lischer, 2010). The partition of genetic variance within and among populations and among genetic groups (determined by Bayesian analysis) was performed through Analysis of Molecular Variance (AMOVA) in ARLEQUIN version 3.5.1.2.

Considering that null alleles were observed in eight loci and that estimates of the inbreeding coefficient (*F*_IS_) and its significance can be biased by the presence of null alleles, we estimated a corrected inbreeding coefficient [*F(Nfb)*] using a model that includes, simultaneously, the parameters null alleles (N), inbreeding coefficients (*F*_IS_), and genotyping failures (B) in a bayesian framework. we performed 500,000 Steps, with 50,000 steps as burn-in and keeping every 100^th^ step for each population using INEST version 2.2 (Chybicki and Burczyk, 2009).

To evaluate the pairwise genetic differentiation between populations *R*_ST_ statistics (**Supplementary Table 2**) were computed using ARLEQUIN version 3.5.1.2 (Excoffier and Lischer, 2010) with significance obtained using 10,000 randomizations and a Bonferroni correction. To verify the correlation between genetic differentiation and geographic distance (IBD), a Mantel test was performed in ARLEQUIN version 3.5.1. We also tested the presence of variation in the IBD effect along the sampled area using the software localDiff (Duforet-Frebourg and Blum, 2013).

To infer *Q. grandiflora* genetic structure, we estimated the most probable number of genetic clusters (*K*) in the total sample by Bayesian analyses using the software STRUCTURE version 2.3.3 (Pritchard et al., 2000). Ten independent runs were performed for *K* varying from one to 24, each with 500,000 Markov chain Monte Carlo (MCMC) replicates and burn-in of 100,000 replicates, assuming an admixture model with correlated alleles between populations. The most probable *K* explaining the data was determined from average likelihood values (‘log of probability’, [LnP (D)]) across runs for each *K*, as suggested by Pritchard et al. (2000), and the Δ*K* statistic was calculated according to Evanno et al. (2005). Since the inference of *K* from STRUCTURE can be sensitive to the presence of isolation by distance (IBD), we also inferred genetic clustering considering the geographic localization of the populations, with the software TESS 2.3.1 (Chen et al., 2007). The BYM admixture model was used with default parameters and 200,000 MCMC replicates after 20,000 steps of burn-in. The best *K* was assessed using the Deviance Information Criterion, DIC, and the DK statistic. In addition, we inferred the influence of past climatic changes through a comparison of genetic structure with that revealed with cpDNA and nDNA sequence data in a previous phylogeographic study (Buzatti et al., 2018). A similar spatial genetic structure among the markers would be evidence of the maintenance of gene flow barriers across the time and of the influence of past climatic changes.

### Leaf Metamer Trait Measurements and Analyses

For morphometric evaluation we sampled three fully developed foliar metamers (i.e. internode, petioles, and the corresponding pair of leaf lamina) of the second node from distal branches of different parts of the crown of 744 individuals from 36 populations (totaling 2590 leaves). All samples were collected in the rainy season and the distance between sampled populations ranged from 5.33 to 2,067 km (**Supplementary Table 1**). Voucher specimens of sampled populations were deposited at the herbaria of the Universidade Federal de Minas Gerais (BHCB) and one of them at the Universidade Estadual do Oeste do Paraná (UNOP) (**Supplementary Table 2**).

Once collected, metamers were immediately photographed with a millimeter scale for subsequent measurement in the laboratory of leaf area (LA in cm^2^), leaf length (LL, in cm), leaf width (LW, in cm) and leaf left-side and right-side width (in cm), using the software Image J. All samples were put in paper bags and dried in an oven at 70 °C for 72 h for determination of dry mass of each part of the metamer, separately (leaf mass, LM; petiole mass, PM; and internode mass, IM; all in grams). From these data we estimated the following metamer traits: Internode Mass Ratio (IMR), Petiole Mass Ratio (PMR), Leaf Mass Ratio (LMR), Specific Leaf Area (SLA), Leaf Area Ratio (LAR), Leaf Length per Leaf Width (LLW), Leaf Fluctuating Asymmetry (LFA) and Metamer Fluctuating Asymmetry (MFA). The traits were selected due their physiological importance. Metamer mass (MM), IMR and PMR indicate the biomass investment in biomechanical and hydraulic support (Poorter and Rozendaal, 2008). LMR, SLA and LAR reflect the biomass investment at the metamer level in leaf display and light capture (Poorter and Rozendaal, 2008). LA, LLW and SLA are related to tradeoff between carbon uptake by photosynthesis and water loss by transpiration (Jacobs, 1999; Ackerly et al., 2000; Poorter, 2009; Wiemann et al., 1998). Fluctuating Asymmetry (related to LFA and MFA), is defined as small and random bilateral symmetry and is indicative of ecological stress to which the plant may have been subjected to; in general this is correlated with environmental conditions of temperature, nutrients, light and water (Hódar, 2002). For formula descriptions of and references to the metamer traits used in our analyses, see **Table 2**.

**Table 2:** Metamer traits of *Qualea grandiflora* used in the study and their respective abbreviations, formulas and references.

Metamer traits (unit)	Abbreviation	Formula	References
Leaf area (cm^2^)	LA	Measured using Image J software	Poorter and Rozendaal, 2008
Metamer Mass (g)	MM	Measured using Image J software	Poorter and Rozendaal, 2008
Internode Mass Ratio	IMR	Internode Mass/Metamer Mass	Poorter and Rozendaal, 2008
Petiole Mass Ratio	PMR	Petiole Mass/Metamer Mass	Poorter and Rozendaal, 2008
Leaf Mass Ratio	LMR	Leaf Mass/Metamer Mass	Poorter and Rozendaal, 2008
Specific Leaf Area (cm^2^/g)	SLA	Area of the leaf blade by dry mass unit	Poorter and Rozendaal, 2008
Leaf Area Ratio (cm^2^/g)	LAR	Leaf Area/Metamer Mass	Poorter and Rozendaal, 2008
Leaf Length/Leaf Width	LLW	Leaf Length/Leaf Width	Wiemann et al., 1998; Jacobs, 1999
Leaf Fluctuating Asymmetry	LFA	(Leaf Left-side Width – Leaf Right-side Width)/Leaf Total Width	Hódar, 2002
Metamer Fluctuating Asymmetry	MFA	(Left Leaf Width – Right Leaf Width)/Leaves Total Width	Hódar, 2002

Similar to the partitioning of genetic diversity, we partitioned the metamer trait variation into three hierarchical levels in order to analyze the proportion of variance found among populations, among individuals within populations and among metamers within individuals. We performed a linear mixed effects models using the R package ‘nlme’ (Pinheiro et al., 2014; R Core Team, 2018). The hierarchical levels were defined as nested random effects and the final level was the error term and can be thought of as partly due to phenotypic plasticity, since all metamers of the same individual have the same genotype. For each metamer trait, we compared the observed variance at each level with the variance calculated after randomizing the data; the data were randomized 999 times to create a random distribution with which to compare to the observed variance and to calculate *P* values.

### Drivers Underlying Genetic and Metamer Trait Divergence and Diversity

In order to test the relative importance of environment factors and geographic distance in shaping the genetic and metamer trait divergence among populations in *Q. grandiflora* we performed Generalized Dissimilarity Modelling (GDM), with the R package ‘gdm’ (Manion et al., 2018; R Core Team, 2018). GDM is a permutational matrix regression that uses *I-*spline functions to model nonlinear relationships between biological, environmental and geographic variables (Ferrier et al., 2007; Fitzpatrick and Keller, 2015; Oliveira et al., 2018). In this study, two GDM models were fit. In the first one, for the genetic data, we used a pairwise R_ST_ matrix (**Supplementary Table 3**) as the response variable while in the other model, we used a Mahalanobis pairwise distance matrix generated from the metamer trait data as the response variable. In both models, geographic distance between populations and environmental variables (climate, soil and wind speed) were used as predictor variables.

The climate variables and wind speed for each population were extracted from WorldClim (Fick and Hijmans, 2017) and soil variables from various sources (**Table 3**) (Dunne and Willmott, 2000; Hengl et al., 2017). We removed strongly correlated variables (*r* > 0.80), retaining eight bioclimatic variables, wind speed, and six soil variables as predictors (**Table 3**). The choice of the climatic variables was based both on previous studies that investigated the correlation between climate, leaf traits (Ribeiro et al., 2016b; Souza et al., 2018) and genetic diversity (Ribeiro et al., 2016b) in the Cerrado and taking into account the known seasonality of the Cerrado, which has a well-defined dry season. The choice of soil variables was made based on those related to nutrient (e.g., soil pH, cation exchange capacity) and water (plant extractable water capacity) availability and also on variables previously known to affect functional traits, such as soil bulk density (Maire et al., 2015). For the genetic data model, the wind speed variable consisted of the mean of the wind speed during the months of species seed dispersal (August-September; Lorenzi, 1992), since *Q. grandiflora* is an anemochoric species (Oliveira et al., 2004). For the metamer trait data model, the mean of wind speed was calculated over the months of the metamer growth (September-November; Lorenzi, 1992). Since the climate maps were at a resolution of 30 seconds (∼1 km^2^) and the soil maps were at a resolution of 250 m, we used the function resample from the ‘raster’ package in R (Hijmans 2017; R Core Team, 2018) to make the resolutions equivalent. After obtaining the final GDM models, a backward stepwise matrix regression was performed with 1000 permutations eliminating minor explanatory variables (based on I-spline values) at each step. The procedure was repeated until all variables retained in the model were significant (*P* < 0.05).

**Table 3:** Predictor variables used in Generalized Dissimilarity Model (GDM) and multiple regression analyses.

Code	Predictor variables	Base data (Reference)
Bio2	Mean Diurnal Range (Mean of monthly (max temp – min temp))	Worldclim (Fick & Hijmans, 2017)
Bio4	Temperature Seasonality (standard deviation *100)	Worldclim (Fick & Hijmans, 2017)
Bio8	Mean Temperature of Wettest Quarter	Worldclim (Fick & Hijmans, 2017)
Bio9	Mean Temperature of Driest Quarter	Worldclim (Fick & Hijmans, 2017)
Bio16	Precipitation of Wettest Quarter	Worldclim (Fick & Hijmans, 2017)
Bio17	Precipitation of Driest Quarter	Worldclim (Fick & Hijmans, 2017)
Bio18	Precipitation of Warmest Quarter	Worldclim (Fick & Hijmans, 2017)
Bio19	Precipitation of Coldest Quarter	Worldclim (Fick & Hijmans, 2017)
s1	Soil organic carbon content (g Kg^−1^)	ISRIC – World Soil Information (Hengl et al., 2017)
s3	soil pH x 10 in KCl – to convert to pH value divide by 10	ISRIC – World Soil Information (Hengl et al., 2017)
s4	Cation exchange capacity (CEC) (cmol Kg^−1^)	ISRIC – World Soil Information (Hengl et al., 2017)
s5	Derived available soil capacity (volumetric fraction) until wilting point – (v%)	ISRIC – World Soil Information (Hengl et al., 2017)
s6	Bulk density of the fine earth fraction (< 2mm; Kg m^−3^)	ISRIC – World Soil Information (Hengl et al., 2017)
s7	Plant extractable water capacity (cm)	DAAC (Dunne and Willmott, 2000)
wind	wind speed (m s^−1^)	Worldclim (Fick & Hijmans, 2017)

In addition to GDM, in order to investigate the association between each metamer trait and the environmental variables, we performed a multiple regression analysis using the mean of the traits of each population as the response variable. We used a stepwise model selection routine from the function ‘step’ implemented in the ‘stats’ package in R (R Core Team, 2018) in order to select the model with minimum value of the Akaike Information Criterion (AIC). Posteriorly, we manually removed from the final model the variables with significance level > 0.05 and variance inflation factor (VIF; function ‘vif’ of the ‘car’ package in R) >5 (Fox and Monette, 1992; Fox and Weisberg, 2011; Fox, 2016; R Core Team, 2018).

Finally, to test for an association between genetic and metamer traits divergence, we performed a multiple regression on distance matrices with the function ‘mrm’ from ‘ecodist’ package in R (Goslee and Urban, 2007; R Core Team, 2018), using pairwise *R*_ST_ and pairwise Mahalanobis distance matrices.

## Results

### Genetic Diversity, Structure, and Drivers of Genetic Divergence

The total number of alleles across the nine microsatellite loci analyzed was 219, with 24.3 alleles per locus, on average. The genetic diversity was heterogeneous over the populations, with the mean number of alleles per locus (*N*_A_) varying from 6.3 to 10.9, allelic richness (*A*_R_) varying from 5.1 to 7.7, observed heterozygosity (*H*_O_) and expected heterozygosity (*H*_E_) ranging from 0.42 to 0.77 and 0.69 to 0.85, respectively (**Table 1**). Populations Chapada dos Guimarães (CHG), Pirinópolis (PIR), and Nova Xavantina (NXA) showed higher *A*_R_ and *N*_A_ while populations PIR, Caldas Novas (CAL), Corinto (COR), Campo Grande (CQG), and CHG showed higher *H*_E_ (**Table 1**). The lowest *A*_R_ and *H*_E_ were found in the Amazonian savanna populations (HTA, SAN) and peripheral northeastern population PRI. The *F(nfb)* was significant (*P* < 0.05) in seven populations, ranging from 0.029 (PIR) to 0.189 (FUR) (**Table 1**). The majority of the genetic variation was found among populations (*R*_ST_ = 0.177; **Table 4**).

**Table 4:** Results of the analysis of molecular variance (AMOVA) for *Qualea grandiflora*.

Source of variation	Variance component	Percentage of variance	Fixation indexes (*P*)
**Genetic variation**			
Among populations	37.78	17.7	*R*_ST_ = 0.177 (<0.0001)
Within populations	176.19	82.3	
**Genetic variation including Bayesian groups**			
Among groups	0.18	4.7	*F*_CT_ = 0.047
Among populations within groups	1.2	5.3	*F*_SC_ = 0.055
Within populations	3.38	90.1	*F*_ST_ = 0.099

**Figure 1:**
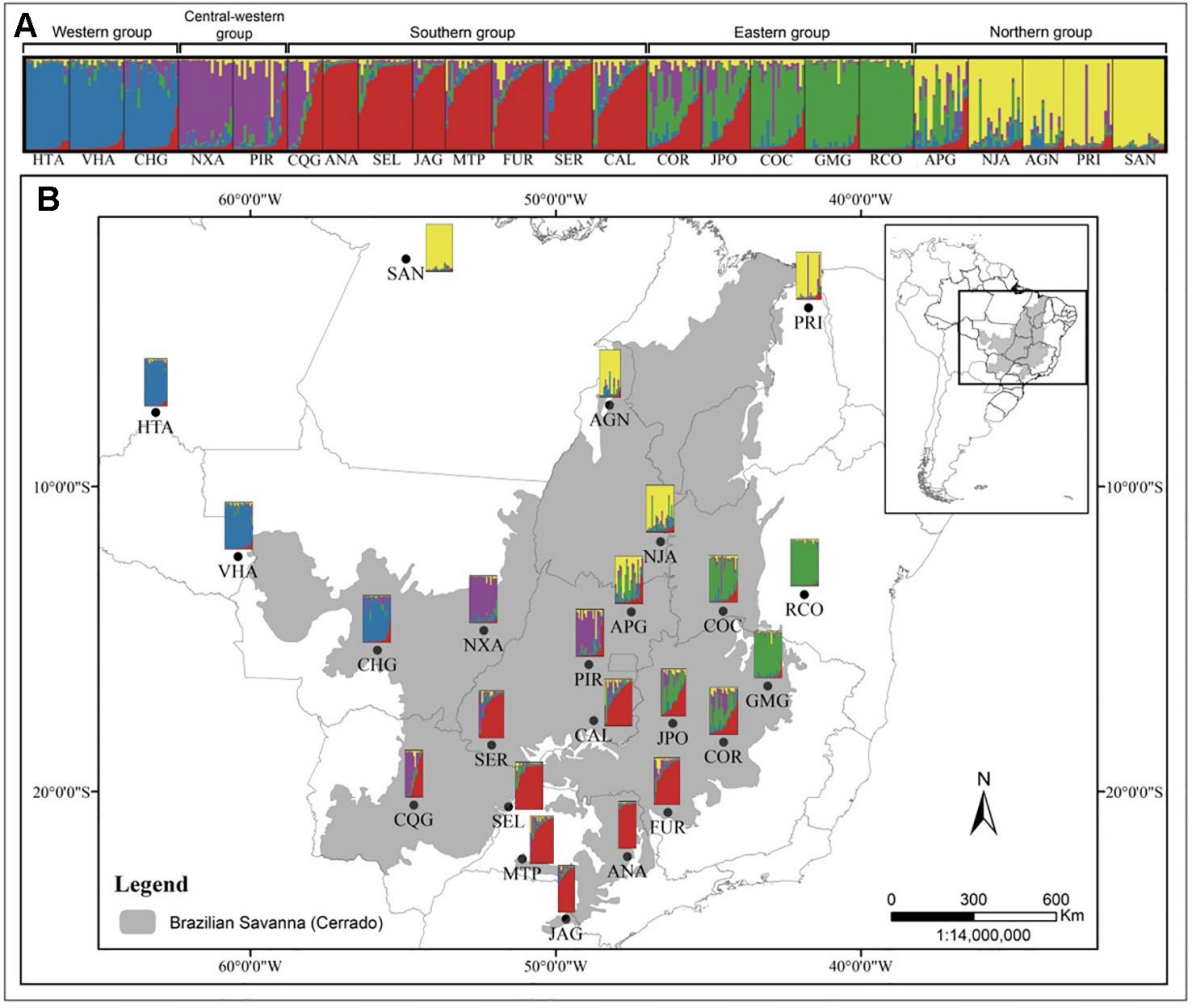
Barplot representation of the five genetic groups of *Qualea grandiflora* inferred by Bayesian analysis (STRUCTURE) of 418 individuals from 23 populations **(A)**. Map showing the sampled populations of *Q. grandiflora* and its population structure as determined by STRUCTURE **(B)**.

The Mantel test showed a high, positive correlation between genetic [*R*_ST_/(1 – *R*_ST_)] and geographic distances (*r* = 0.833; *r*^2^ = 0.694; *P* < 0.001), indicating IBD. The test performed with the program localDiff showed that genetic differentiation between populations seems to remain constant across sampled area since the stationary hypothesis was not rejected (*P* > 0.05). Structure inferences, both from STRUCTURE and TESS analyses resulted in *K* = 5 genetic groups, geographically structured (**Figure 1** and **Supplementary Figure 2**, respectively). This structure was similar to that found by Buzatti et al. (2018) with cpDNA and nuclear sequences. The genetic diversity within each group was similar in relation to *H*_E_, but was different for *A*_R,_ with the central-western group showing the highest value (15.5) and the northern group the lowest value (12.0) (**Table 1**). The populations APG, CQG and JPO and COR, which are located in the boundary among the groups showed high levels of genetic admixture (**Figure 1**). A hierarchical AMOVA including Bayesian groups revealed that the variation among the five groups defined by STRUCTURE was significant and explained a considerable part of total variation found among populations (*F*_CT_ = 0.047, *P* < 0.001; **Table 4**).

**Figure 2:**
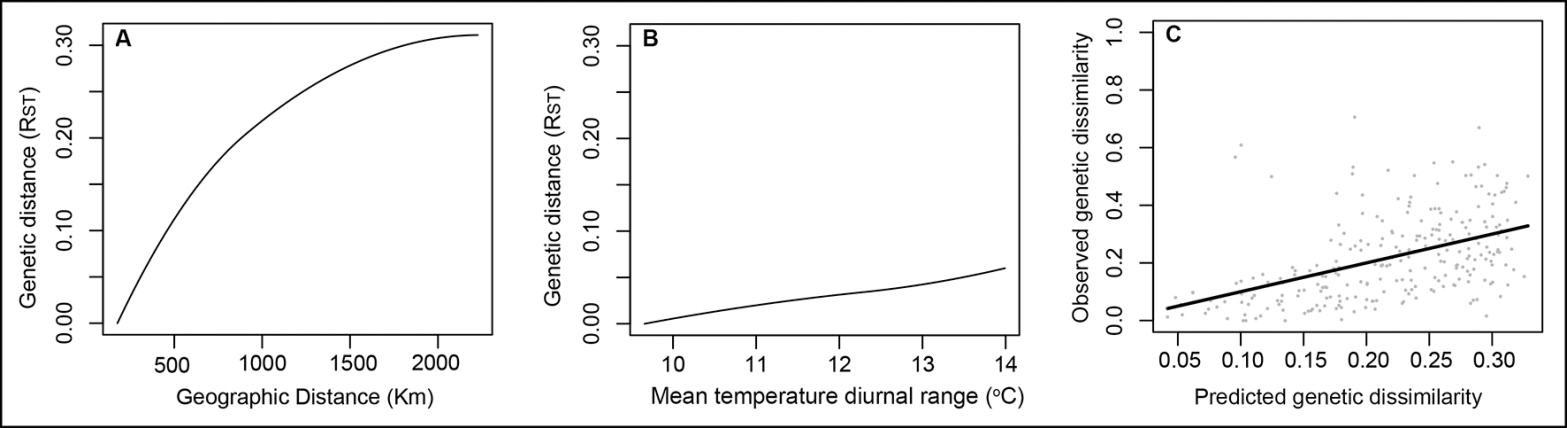
GDM-fitted I-splines for the geographic distance **(A)** and mean temperature diurnal range **(B)** associated with genetic differentiation in *Qualea grandiflora*. The maximum height of each curve indicates the total amount of genetic differentiation associated with that variable holding all other variables constant and the curve shape shows how the rate of genetic differentiation varies along the gradient. **(**Panel **C)** illustrates observed versus predicted genetic dissimilarity.

The complete GDM model for genetic data explained 36.7% of the total observed genetic variation. Six variables showed *I*-splines equal to zero and were not included in the model. Only geographic distance and mean diurnal temperature range (Bio2) were significant and remained in the model (*P* < 0.05). The GDM final model was then composed by only these two variables, which explained 23.1% of the total variance. Geographic distance was the most important variable in the model (*I*-spline = 0.31), showing its strong influence in the genetic divergence among populations and corroborating the Mantel test. Mean diurnal temperature range had lower influence on the genetic divergence among populations (*I*-spline = 0.06) (**Figure 2**).

### Metamer Trait Variation and Drivers

**Figure 3:**
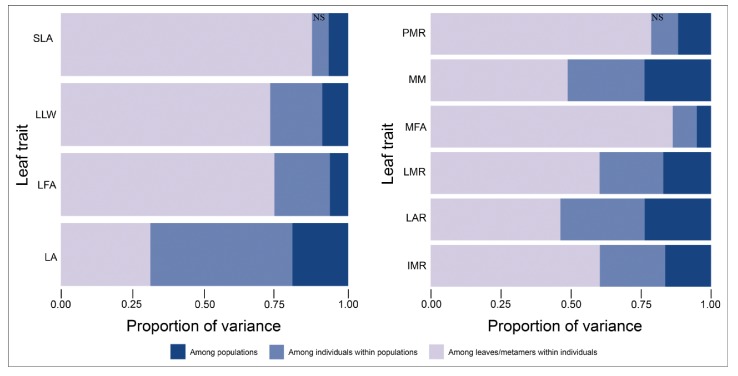
Hierarchical partitioning of variance for metamer traits in *Qualea grandiflora*. Variance components and significance levels were determined by randomization (see Methods). All variance components were significant, except SLA and PMR among individuals (NS).

The linear mixed effects models showed significant differences among populations for all leaf traits, representing 5 to 24% of the total variation, depending on the trait (**Figure 3**). Most variation however, was found among leaves/metamers within individuals (31–87%) and among individuals within populations (6 to 50%). Differences among individuals within populations were significant for all traits, except for SLA and PMR. More than 75% of total variation in the traits SLA, PMR and MFA was due to variation among leaves/metamers within individuals. LA, MM, and LAR were the traits that showed the highest percentage of variation among populations (**Figure 3**).

**Figure 4:**
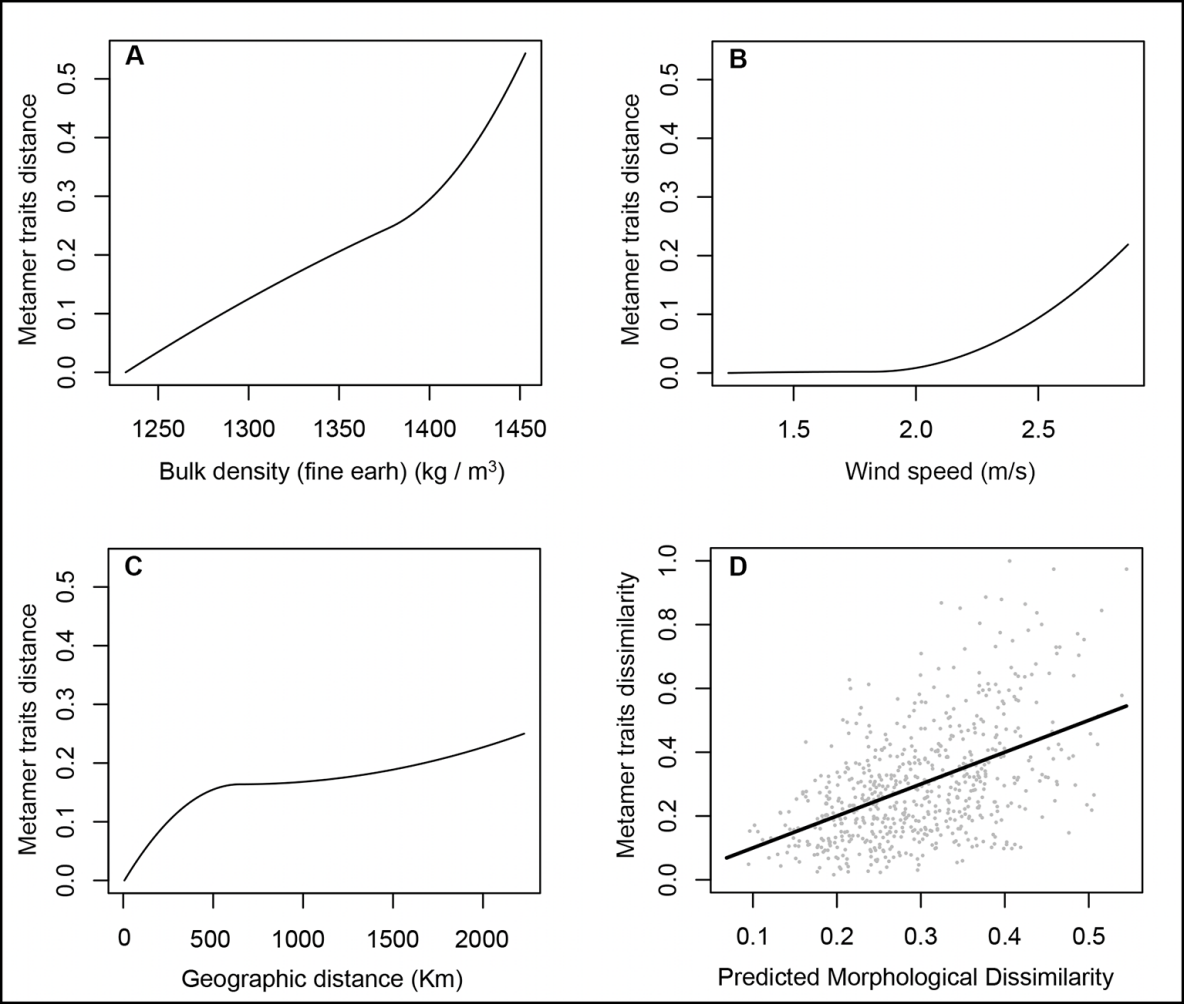
GDM-fitted I-splines for the bulk density **(A)**, wind speed **(B)** and geographic distance **(C)** associated with metamer trait dissimilarity in *Qualea grandiflora*. The maximum height of each curve indicates the total amount of metamer differentiation associated with that variable holding all other variables constant and the curve shape shows how the rate of metamer trait differentiation varies along the gradient. **(**Panel **D)** illustrates observed versus predicted metamer trait dissimilarity.

GDM analysis using a Mahalanobis distance matrix of leaf traits as the response variable explained 32.5% of the total model deviance. Four of the 15 environmental predictors were not important for the model and showed *I*-spline values equal to zero but only soil bulk density and wind speed made significant contributions (*P* ≤ 0.05) in addition to geographic distance. So, the final model to describe the metamer trait dissimilarity in *Q. grandiflora* includes soil bulk density (s6; *I*-spline = 0.55), wind speed (*I*-spline = 0.22) and geographic distance (*I*-spline = 0.25), which explains 24.9% of the total metamer trait variation among populations (**Figure 4**).

Multiple regression analyses showed that climate and edaphic variables explained a large proportion of the metamer trait variation among populations, with most of the models explaining more than 50% of the variation (**Table 5**). Of the 10 analyzed traits, most (seven traits) were associated with both climate and soil variables and three (LA, LLW and MFA) were associated only with climate variables. Four soil variables influenced the metamer traits: soil pH in KCl (s3), derived available soil water capacity (s5), bulk density (s6) and plant extractable water capacity (s7). Bulk density was the soil variable associated with the greatest number of phenotypic traits (four) followed by derived available soil water capacity (three). Six out of eight climate variables affected the traits (two temperature and four precipitation variables). Precipitation of the wettest quarter (Bio16) and precipitation of warmest quarter (Bio18), which represent the quarters with highest precipitation during the year (except in locations of the northernmost populations SAN, CAG and AGN), were the climate predictors associated with most of the metamer trait variation. Bio16 or Bio18 were predictors for all 10 traits investigated (**Table 5**). Mean diurnal temperature range (Bio2), mean temperature of the wettest quarter (Bio8) and precipitation of the driest quarter (Bio17) affected two traits each and precipitation of coldest quarter (Bio19) affected one. Variation in four metamer traits (LA, MM, LMR, IMR) was also associated with mean wind speed during their development (**Table 5**).

**Table 5:** Results of multiple regression analyses (intercept and β coefficient values for significant variables; *P* < 0.05) relating *Qualea grandiflora* metamer traits with geographic and environmental variables.

Morphological traits	Intercept	Environmental variables													
		Geography	Temperature		Precipitation				Soil						
		LONG	BIO2	BIO8	BIO16	BIO17	BIO18	BIO19	S3	S5	S6	S7	WIND	AIC	R^2^
Leaf area (LA) (cm^2^)				4.0176			0.0919						–16.9914	273.5	0.506
Leaf Length/Leaf Width (LLW) (cm)	2.0580					0.0024	5.06E–04	3.15E–04						–38.4	0.644
Leaf Fluctuating Asymmetry (LFA) (cm)	0.2617		–0.0103		–9.34E–05							–2.42E–03		–160.0	0.340
Specific Leaf Area (SLA) (cm^2^/g)					–0.0314	0.0842				–1.6631	0.1015			260.4	0.558
Metamer Mass (MM) (g)				0.2025			5.03E–03			0.0905		–0.0520	–0.8107	35.8	0.730
Petiole Mass/Internode Mass Ratio (PMR) (g)		–4.25E–04			–1.08E–05		1.83E–05		1.11E–03					–301.0	0.491
Leaf Mass/Metamer Mass Ratio (LMR) (g)	0.6726				–6.00E–05		4.80E–05				2.04E–04		–0.0297	–207.5	0.638
Internode Mass/Metamer Mass Ratio (IMR) (g)	0.2761				6.73E–05		–6.31E–05				–1.84E–04		0.0294	–216.0	0.650
Metamer Fluctuating Asymmetry (MFA) (cm)	0.1472		–5.27E–03		–4.68E–05									–213.7	0.275
Leaf Area Ratio (LAR) (cm^2^/g)					–0.0306					–1.1296	0.0646			237.1	0.542

^a^LONG, Longitude; BIO2, Mean Diurnal Range; BIO8, Mean Temperature of Wettest Quarter; BIO16, Precipitation of Wettest Quarter; BIO17, Precipitation of Driest Quarter; BIO18, Precipitation of Warmest Quarter; BIO19, Precipitation of Coldest Quarter; S3, Soil pH x 10 in KCl; S5, Derived available soil capacity; S6, Bulk density; S7, Plant extractable water capacity; WIND, Wind speed.

Leaf area (LA) and metamer mass (MM) were associated with variables that provide higher plant productivity. They were positively associated with precipitation of the warmest quarter (Bio 18) and temperature of wettest quarter (Bio8), but negatively associated with wind speed (**Table 5**). Metamer Mass (MM) also had an association with soil water capacity (s5). SLA was positively related to precipitation of the driest quarter (Bio17) and negatively related to precipitation of the wettest quarter (Bio16), indicating that there is a decrease of SLA in populations of more arid climates (**Table 5**). In the most arid parts of the Cerrado, precipitation is concentrated into only a few months. The other trait associated with light capture, leaf area ratio of metamer (LAR), was also negatively associated with Bio16, also suggesting a lower LAR in more arid climates. In addition, SLA and LAR are negatively related to derived available soil water capacity (s5) and positively related to bulk density (s6; **Table 5**). The proportion of metamer mass that is invested in leaf (LMR; Leaf Mass/Metamer Mass) and petiole (PMR; Petiole Mass/Metamer Mass) had a complex association with environmental predictors, decreasing with the increase of precipitation of the wettest quarter (Bio16) and increasing with precipitation of the warmest quarter (Bio18). LMR also had a negative association with wind speed. The proportion of metamer mass invested in internode (IMR) was influenced by the same variables of LMR, but in inverse direction, i.e. the conditions that increase LMR decrease IMR. Fluctuating asymmetry of both leaf and metamer was negatively associated with diurnal temperature range (Bio2) and with precipitation of wettest quarter (Bio16). So, populations in sites with higher water limitation had higher fluctuating asymmetry (**Table 5**). The results of the association test between the pairwise *R*_ST_ and pairwise Mahalanobis distance matrices showed no relationship between metamer traits and genetic divergence (r = 0.06; *r*^2^ = 0.004; *P* = 0.848).

## Discussion

Our study of the most widely distributed tree species in the Cerrado biome, *Q. grandiflora*, sampled across its entire distribution, revealed significant variation within and among populations for both genetic data and phenotypic metamer traits. As we hypothesized, environmental factors and geography play distinct roles in the species’ intraspecific diversity. While climate and soil contribute more to leaf trait variation, geography seems to contribute substantially to genetic variation. Isolation by distance (IBD) appeared to be the main determinant of genetic divergence among populations, although IBE also had a limited effect. Soil bulk density and wind speed during the period of metamer development were important drivers of metamer trait divergence among populations along with geographic distance. Although soil factors explained the variation in several metamer traits, climate (mainly precipitation) was related to all metamer traits.

GDM results suggesting geographic distance as the main factor promoting genetic divergence among *Q. grandiflora* populations was corroborated by the Mantel test that found a strong correlation between genetic and geographic distances (*r* = 0.833). In addition, our results also suggested that differences in the mean diurnal temperature range of sampled sites also contribute towards explaining the genetic differences among populations. Although geographic distance has been emphasized as the most common factor limiting gene flow among populations, recent landscape genetic studies suggest that this factor is just one of many possible causes of genetic divergence and isolation and that environmental heterogeneity may also play an important role in this process (Bond et al., 2014; Ribeiro et al., 2016b; Oliveira et al., 2018). While microsatellites are considered neutral markers, the IBE found in *Q. grandiflora* could be a reflection of the effect of selection at loci that are closely linked to the studied loci (i.e., a hitchhiking effect, Barton, 2000). An IBE effect determined by climate variables was found as the main factor shaping genetic divergence in another tree species from the Cerrado, *Annona crassiflora* (Annonaceae, Ribeiro et al., 2016b). Despite *Q. grandiflora* showing lower genetic divergence among populations and thereby revealing higher gene flow than what has been found in *A. crassiflora*, the divergence seems to be mainly explained by IBD. The difference in genetic divergence between the two species could be due to differences in seed dispersion mechanisms between them. Seed dispersal in *Q. grandiflora* is anemochoric (Antiqueira and Kageyama, 2014), whereas seed dispersal in *A. crassiflora* is compromised, since large mammals that were its main dispersers went extinct during the early Holocene (Ribeiro et al., 2016b).

Our study of *Q. grandiflora* indicated soil bulk density as an important predictor, in addition to wind speed and geographic distance, of the phenotypic divergence among populations for all metamer traits considered together. Bulk density is an indicator of soil compaction and porosity that affect the available water capacity, water movement through soil and root growth (www.nrcs.usda.gov/Internet/FSE_DOCUMENTS/nrcs142p2_053260.pdf). Edaphic factors have been pointed out as mainly responsible for promoting distinct floristic composition even under similar climate conditions in the Cerrado biome (Furley and Ratter, 1988; Ruggiero et al., 2002; Ratter et al., 2003; Assis et al., 2011; Bueno et al., 2018). However, the role of soil in shaping intra-specific divergence in functional traits of Cerrado plants is poorly known. Wind has multiple effects on plants. It can promote mechanical damage, and affect the heat, gas exchange and transpiration by affecting the thickness of the boundary layer, the air layer adjacent to the leaf. The thickness of the boundary layer depends on leaf characteristics and wind speed. Larger leaves subject to the same wind speed have higher boundary layer thickness (Grace, 1988; Anten et al., 2010).

The associations of each metamer trait with the 15 tested environmental variables (including geographic, climate and edaphic characteristics) in *Q. grandiflora* were complex. However, some clear patterns could be identified by the multiple regression analysis. The traits associated with light capture, such as SLA and LAR were lower in populations in drier climates. Populations located in sites with climate conditions favorable to plant productivity, such as sites with high precipitation and high summer temperatures, showed larger leaves and heavier metamers. Soils with higher available water capacity also favored metamer size. The investment in leaf mass (LMR) was affected by precipitation in the quarters with highest rainfall during the year, and was also higher in populations located in areas with less wind. A trade-off seems to exist between the investment in leaf mass (LMR) and the investment in internode mass (IMR) in *Q. grandiflora*, since they were affected by the same environmental variables, but in opposite directions. Internode mass is important for biomechanical leaf sheet support and the hydraulic pathway (Santiago et al., 2004; Souza et al., 2018). In *Q. grandiflora*, we found higher fluctuating asymmetry in metamers and leaves in populations from sites with lower precipitation in the rainy season and lower plant extractable water capacity, respectively. This result is consistent with a relationship between fluctuating asymmetry and stressful conditions sometimes documented in plants (Hódar, 2002; Lobregat et al., 2018; Tucić et al., 2018).

Soil characteristics show even more complex relationships with the metamer traits than climate variables. Bulk soil density was positively related to SLA, LMR and LAR, but negatively related to IMR. These relationships of metamer traits with soil bulk density need to be better studied, since they are apparently contradict theoretically expectations. It is known that high soil bulk density affects plant growth (Houlbrooke et al., 1997). The influence of physical soil properties on leaf traits in wild species is poorly known in comparison with climate (but see Maire et al., 2015), and we did not find any study regarding the effects of physical soil properties on Cerrado tree species in the literature.

Our data indicate that wind speed is an important environmental factor influencing metamer trait divergence in *Q. grandiflora*, since the GDM analysis revealed an association with the overall metamer trait divergence among populations and the multiple regression showed its association with four specific metamer traits. Populations in environments with higher wind speed had smaller LA, MM and LMR, and higher IMR. Our results are concordant with some studies that reported reductions in LA and SLA and an increase in leaf thickness as morphological responses of plants to higher wind speeds (Wu et al., 2016; Zhang et al., 2018). Moreover, strong winds can lead to shorter and thicker internodes, promoting more mechanical resistance against wind (Huber et al., 2014). The positive association between IMR and wind speed found in *Q. grandiflora* can be due the internode thickening or due to the decrease of MM in areas with faster wind. Moreover, the decrease of MM in populations located in regions with higher wind speed could also be related to the reduction of LA and leaf thickness. Wind speed has been rarely investigated as a factor that can influence metamer functional traits in wild species. Our results indicate its importance in shaping metamer trait divergence in a typical species of the Cerrado suggesting that it should not be neglected.

The partitioning of phenotypic metamer variation showed that for all traits, except LA, most of the variation is found within individuals. This result is in accordance with those found in another widely distributed tree in the Neotropics, *Copaifera langsdorffii*, which revealed considerable phenotypic plasticity in the species (Souza et al., 2018). Phenotypic plasticity is the ability of a single genotype to produce different phenotypes when exposed to different environmental conditions (Sultan, 2000; Auld et al., 2010). The high variation within individuals of *Q. grandiflora* suggests that a considerable part of the total phenotypic variation in metamer traits can be attributed to phenotypic plasticity. In fact, considerable phenotypic plasticity has been found for leaf traits in Cerrado trees (Goulart et al., 2011; Barros et al., 2012; Souza et al., 2018). Phenotypic plasticity is probably an important factor in allowing *Q. grandiflora* to reach nearly all parts of the Cerrado, encompassing a great variety of environmental conditions. However, the genetic variation among individuals and populations also likely contributes to the high phenotypic variation in metamer traits found in the species. Although local adaptation may create associations between phenotypes and environmental gradients, especially in plants occurring across heterogeneous landscapes (Goulart et al., 2006; Nakazato et al., 2008; Goulart et al., 2011; Toledo et al., 2012; Ribeiro et al., 2016b), an extensive investigation is necessary before concluding that they represent adaptations (Gould and Lewontin, 1979). Thus, an evaluation of the relative contribution of local adaptation and phenotypic plasticity for the metamer trait variation in *Q. grandiflora* could be assessed through analysis of these traits in plants grown in common gardens (e.g. Goulart et al., 2011; Barros et al., 2012).

*Q. grandiflora* populations exhibited high genetic variation, with populations located at the central portion of the Cerrado core (PIR, CAL, COR, and CQG) showing the highest genetic diversity. Moreover, the populations located in this central portion showed high admixture levels. These results are consistent with the colonization history of *Q. grandiflora*, which revealed that the central portion of the Cerrado core was the species’ refuge during the cold ages of the Quaternary Period (Buzatti et al., 2018). From 23 analyzed populations, 7 (30%) showed significantly positive *F*_IS_, suggesting some inbreeding. Studies of breeding systems suggest that *Q. grandiflora* is self-incompatible (Barbosa 1983; Oliveira et al., 2004), although Antiqueira and Kageyama (2014) found a low rate of selfing in a mating system study. However, Oliveira et al. (2004) observed that despite the occurrence of self-fertilization in *Q. grandiflora*, some sort of late-acting self-incompatibility mechanism is present in the species. Thus, the inbreeding coefficient found here in those seven populations may be due to both mating among relatives and small levels of selfing.

The spatial genetic structure found here using microsatellites was similar to the structure revealed with cpDNA and nDNA sequence data (Buzatti et al., 2018), suggesting that ancient gene flow barriers may have been maintained over time. Our study revealed an east-west split in the Cerrado core, as previously reported in several phylogeographic studies of different Cerrado’ species (Novaes et al., 2013; Ribeiro et al., 2016a; Ribeiro et al., 2016b; Resende-Moreira et al., 2017; Buzatti et al., 2018). These studies suggested that the vicariance process is caused by the mountain range present in the region interacting with the Pleistocene climate fluctuations, and specifically for *Q. grandiflora*, reduced gene flow seems to be caused mainly by the Serra Geral plateau, the Central Brazilian plateau and the Canastra mountain range (Buzatti et al., 2018). Additionally, we inferred two other distinct genetic groups in the southern and northern Cerrado, a pattern also observed in the colonization history study of *Q. grandiflora* (Buzatti et al., 2018). Likewise, phylogeographic studies show similar differentiation between northern and southern portions of the Cerrado biome, also attributed to Pleistocene climate change (Novaes et al., 2010; Novaes et al., 2013; Buzatti et al., 2018). Moreover, our results corroborate the isolation among different regions in the Amazon, since SAN and HTA in central and southwestern parts of the Amazon, respectively, were found to belong to distinct genetic groups (see Buzatti et al., 2018; Resende-Moreira et al., 2018).

In summary, our results demonstrate that geographic distance and past climate change shaped the genetic diversity and structure of *Q. grandiflora*. Geographic distance was less important for metamer divergence. Environmental variation across the wide distribution of this species (mainly variation in precipitation, soil bulk density and wind speed) likely plays an important role in promoting divergence in metamer traits. The high variation in metamer traits, which our data suggest are likely due to genetic variation and phenotypic plasticity and can explain the ability of the species to occupy the entire Cerrado, including savanna enclaves in the Amazon. In addition, our data suggest that if considerable areas of the Cerrado are preserved and connected to each other, the high genetic and leaf trait diversity found in this species may allow *Q. grandiflora* to cope with current and future climate change.

## Data Availability Statement

Microsatellite genotypes and leaf trait datasets generated and analysed in this study can be found in the Figshare repository with DOI: 10.6084/m9.figshare.11106881. Leaf trait dataset was also submitted to TRY Plant Trait Database. All other datasets for this study are included in the article/**Supplementary Material**.

## Author Contributions

JL-F and ML planned and designed the research, provided financial resources for the research and collected the population samples. RB and TP performed the laboratory work. RB, TP, AM, VE, and RS analyzed the data. RB, TP, JL-F, and ML interpreted the data. RB, TP, JL-F, and ML wrote the manuscript and all authors reviewed the final version. RB and TP should be considered joint first authors. The authors JL-F and ML should be considered joint senior authors.

## Funding

This work was supported by Fundação de Amparo à Pesquisa do Estado de Minas Gerais (FAPEMIG), Conselho Nacional de Desenvolvimento Científico e Tecnológico (CNPq) and Coordenação de Aperfeiçoamento de Pessoal de Nível Superior (CAPES).

## Conflict of Interest

The authors declare that the research was conducted in the absence of any personal, professional or financial relationship that could constitute a conflict of interest.
