# Supplementary material for: Disentangling the Environmental Factors That Shape Genetic and Phenotypic Leaf Trait Variation in the Tree Qualea grandiflora Across the Brazilian Savanna
Source: Front Plant Sci. 2019 Dec 2;10:1580. doi: 10.3389/fpls.2019.01580 (PMC6900740; doi:10.3389/fpls.2019.01580)
Supplement: Supplementary file 1 [file DataSheet_1.docx]

Supplementary Material

# **Supplementary Figures and Tables**


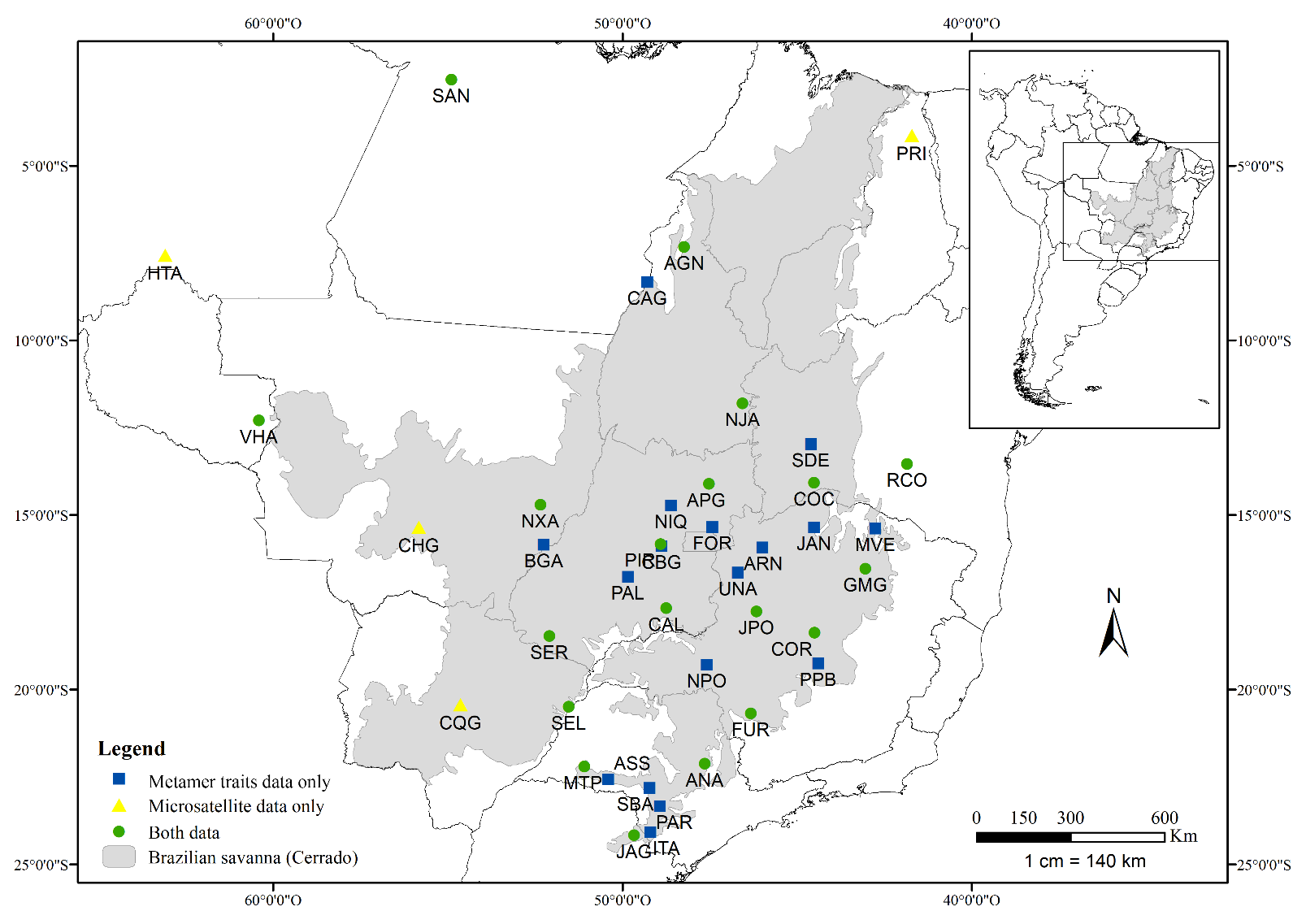


# **Supplementary Figures**

**Supplementary Figure 1.** Map of distribution of *Qualea grandiflora* sampled populations for only metamer traits or microsatellite data or for both data.


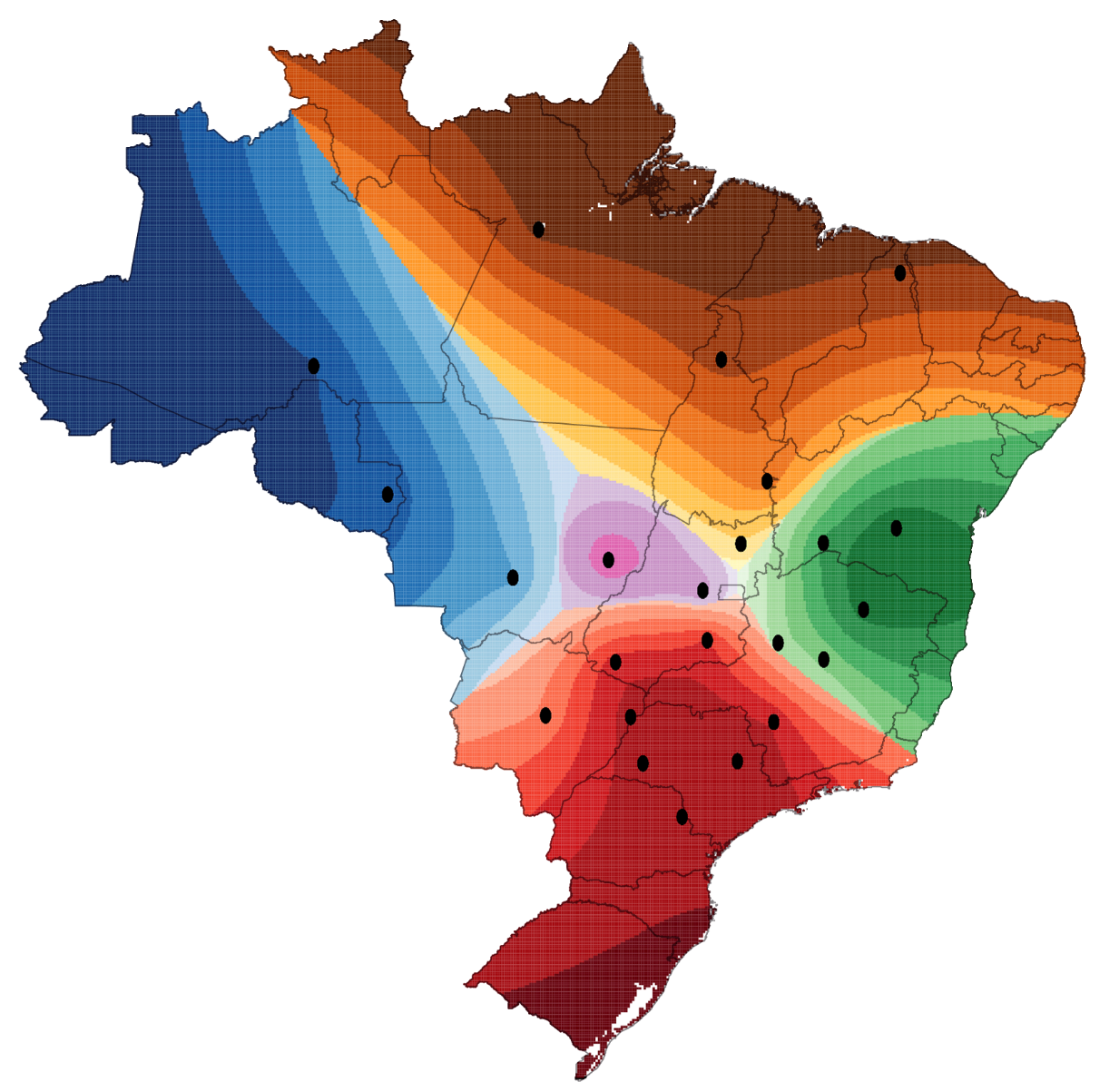


**Supplementary Figure 2.** Graphical representation of the five genetic groups of *Qualea grandiflora* inferred by Bayesian Analyses on TESS.

**1.2 Supplementary Tables**

**Supplementary Table 1.** Matrix of geographical distance among all sampled populations (Km).

**Supplementary Table 2.** List of populations used in this study with herbarium records.

|  |  |
| --- | --- |
| Locality/State^a^ (population code) | Herbarium records |
| Alto do Paraíso de Goiás, GO (APG) | BHCB 147165 |
| Araguaína, TO (AGN) | BHCB174538 |
| Analândia, SP (ANA) | BHCB154272 |
| Arinos, MG (ARN) | BHCB 147158 |
| Assis, SP (ASS) | BHCB147146 |
| Barra do Garças, MT (BGA) | BHCB169631 |
| Conceição do Araguaia, PA (CAG) | BHCB174540 |
| Caldas Novas, GO (CAL) | BHCB 147175 |
| Corumbá de Goiás, GO (CBG) | BHCB147171 |
| Campo Grande, MS (CHG) | BHCB 175363 |
| Campo Grande, MS (CQG) | BHCB 175363 |
| Cocos, BA (COC) | BHCB 147141 |
| Corinto, MG (COR) | BHCB 147138 |
| Formosa, GO (FOR) | BHCB147163 |
| Furnas, MG (FUR) | BHCB 154282 |
| Grão Mogol, MG (GMG) | BHCB 147145 |
| Humaita, AM (HTA) | BHCB 174533 |
| Itararé, SP (ITA) | BHCB147148 |
| Jaguariaíva, PR (JAG) | BHCB 147149 |
| Januária, MG (JAN) | BHCB 147140 |
| João Pinheiro, MG (JPO) | BHCB 147150 |
| Martinópolis, SP (MTP) | BHCB 147152 |
| Mato Verde, MG (MVE) | BHCB 147144 |
| Niquelândia, GO (NIQ) | BHCB 147166 |
| Nova Xavantina, MT (NXA) | BHCB 169629 |
| Nova Ponte, MG (NPO) | BHCB 154285 |
| Novo Jardim, TO (NJA) | BHCB 169626 |
| Palmeiras de Goiás/GO (PAL) | - |
| Paranapanema, SP (PAR) | BHCB 154258 |
| Paraopeba,MG (PPB) | BHCB 154251 |
| Pirenópolis, GO (PIR) | - |
| Piripiri, PI (PRI) | BHCB 169642 |
| Rio de Contas, BA (RCO) | BHCB 147143 |
| Santarém, PA (SAN) | UNOP 7112 |
| Águas de Santa Barbara, SP (SBA) | BHCB 154256 |
| São Desidério, BA (SDE) | BHCB 147142 |
| Selvíria, MS (SEL) | BHCB 154302 |
| Serranópolis, GO (SER) | BHCB 154298 |
| Unaí, MG (UNA) | BHCB 147156 |
| Vilhena, RO (VHA) | BHCB 174529 |
| *Note:* (-) Absence of herbarium record |  |
| ^a^ Abbreviation of Brazilian States: AM, Amazonas; GO, Goiás; MG, Minas Gerais; MS, Mato Grosso do Sul; MT, Mato Grosso; PA, Pará; PI, Piauí; PR, Paraná; RO, Rondônia; SP, São Paulo; TO, Tocantins | |

**Supplementary Table 3.** Matrix of genetic divergence (*R*_ST_) among sampled populations.

|  | AGN | ANA | APG | CAL | CHG | COC | COR | CQG | FUR | GMG | HTA | JAG | JPO | MTP | NJA | NXA | PIR | PRI | RCO | SAN | SEL | SER | VHA |
| --- | --- | --- | --- | --- | --- | --- | --- | --- | --- | --- | --- | --- | --- | --- | --- | --- | --- | --- | --- | --- | --- | --- | --- |
| AGN | 0.000 |  |  |  |  |  |  |  |  |  |  |  |  |  |  |  |  |  |  |  |  |  |  |
| ANA | 0.506 | 0.000 |  |  |  |  |  |  |  |  |  |  |  |  |  |  |  |  |  |  |  |  |  |
| APG | 0.068 | 0.347 | 0.000 |  |  |  |  |  |  |  |  |  |  |  |  |  |  |  |  |  |  |  |  |
| CAL | 0.221 | 0.159 | 0.147 | 0.000 |  |  |  |  |  |  |  |  |  |  |  |  |  |  |  |  |  |  |  |
| CHG | 0.316 | 0.277 | 0.287 | 0.145 | 0.000 |  |  |  |  |  |  |  |  |  |  |  |  |  |  |  |  |  |  |
| COC | 0.130 | 0.220 | 0.056 | 0.040 | 0.204 | 0.000 |  |  |  |  |  |  |  |  |  |  |  |  |  |  |  |  |  |
| COR | 0.102 | 0.126 | 0.045 | 0.036 | 0.150 | 0.000 | 0.000 |  |  |  |  |  |  |  |  |  |  |  |  |  |  |  |  |
| CQG | 0.225 | 0.509 | 0.377 | 0.248 | 0.332 | 0.389 | 0.299 | 0.000 |  |  |  |  |  |  |  |  |  |  |  |  |  |  |  |
| FUR | 0.193 | 0.081 | 0.083 | 0.042 | 0.244 | 0.031 | 0.020 | 0.362 | 0.000 |  |  |  |  |  |  |  |  |  |  |  |  |  |  |
| GMG | 0.078 | 0.372 | 0.034 | 0.156 | 0.186 | 0.047 | 0.070 | 0.335 | 0.174 | 0.000 |  |  |  |  |  |  |  |  |  |  |  |  |  |
| HTA | 0.466 | 0.206 | 0.269 | 0.193 | 0.268 | 0.298 | 0.178 | 0.504 | 0.133 | 0.333 | 0.000 |  |  |  |  |  |  |  |  |  |  |  |  |
| JAG | 0.396 | 0.063 | 0.207 | 0.077 | 0.183 | 0.109 | 0.074 | 0.532 | 0.034 | 0.294 | 0.248 | 0.000 |  |  |  |  |  |  |  |  |  |  |  |
| JPO | 0.119 | 0.184 | 0.089 | 0.026 | 0.259 | 0.016 | 0.020 | 0.256 | 0.007 | 0.130 | 0.247 | 0.127 | 0.000 |  |  |  |  |  |  |  |  |  |  |
| MTP | 0.341 | 0.079 | 0.247 | 0.086 | 0.227 | 0.092 | 0.041 | 0.500 | 0.025 | 0.294 | 0.324 | 0.040 | 0.069 | 0.000 |  |  |  |  |  |  |  |  |  |
| NJA | 0.105 | 0.547 | 0.000 | 0.304 | 0.397 | 0.137 | 0.125 | 0.551 | 0.174 | 0.087 | 0.446 | 0.375 | 0.184 | 0.388 | 0.000 |  |  |  |  |  |  |  |  |
| NXA | 0.522 | 0.244 | 0.279 | 0.174 | 0.147 | 0.261 | 0.191 | 0.707 | 0.143 | 0.429 | 0.215 | 0.060 | 0.278 | 0.196 | 0.441 | 0.000 |  |  |  |  |  |  |  |
| PIR | 0.209 | 0.264 | 0.097 | 0.099 | 0.193 | 0.107 | 0.081 | 0.436 | 0.053 | 0.207 | 0.159 | 0.106 | 0.087 | 0.135 | 0.161 | 0.068 | 0.000 |  |  |  |  |  |  |
| PRI | 0.046 | 0.541 | 0.138 | 0.301 | 0.462 | 0.241 | 0.179 | 0.237 | 0.212 | 0.229 | 0.436 | 0.449 | 0.145 | 0.361 | 0.141 | 0.533 | 0.191 | 0.000 |  |  |  |  |  |
| RCO | 0.065 | 0.411 | 0.051 | 0.189 | 0.214 | 0.066 | 0.053 | 0.378 | 0.191 | 0.000 | 0.439 | 0.324 | 0.159 | 0.298 | 0.115 | 0.476 | 0.244 | 0.240 | 0.000 |  |  |  |  |
| SAN | 0.108 | 0.476 | 0.182 | 0.312 | 0.406 | 0.231 | 0.154 | 0.239 | 0.289 | 0.124 | 0.503 | 0.502 | 0.193 | 0.411 | 0.228 | 0.670 | 0.349 | 0.172 | 0.127 | 0.000 |  |  |  |
| SEL | 0.353 | 0.069 | 0.194 | 0.160 | 0.264 | 0.139 | 0.067 | 0.568 | 0.034 | 0.305 | 0.255 | 0.057 | 0.116 | 0.012 | 0.294 | 0.167 | 0.102 | 0.325 | 0.313 | 0.349 | 0.000 |  |  |
| SER | 0.387 | 0.078 | 0.260 | 0.092 | 0.192 | 0.101 | 0.069 | 0.609 | 0.041 | 0.310 | 0.285 | 0.005 | 0.114 | 0.007 | 0.423 | 0.133 | 0.132 | 0.466 | 0.318 | 0.504 | 0.056 | 0.000 |  |
| VHA | 0.157 | 0.344 | 0.060 | 0.119 | 0.154 | 0.071 | 0.073 | 0.346 | 0.141 | 0.017 | 0.181 | 0.250 | 0.135 | 0.282 | 0.122 | 0.284 | 0.125 | 0.237 | 0.084 | 0.238 | 0.283 | 0.280 | 0.000 |
